# Incidence of antidepressant use among community dwellers with and without Parkinson’s disease – a nationwide cohort study

**DOI:** 10.1186/s12877-021-02145-6

**Published:** 2021-03-23

**Authors:** Eerik Hentilä, Miia Tiihonen, Heidi Taipale, Sirpa Hartikainen, Anna-Maija Tolppanen

**Affiliations:** 1 School of Pharmacy, University of Eastern Finland, P.O. Box 1627, 70211 Kuopio, Finland; 2 Kuopio Research Centre of Geriatric Care, University of Eastern Finland, P.O. Box 1627, 70211 Kuopio, Finland; 3 Department of Clinical Neuroscience, Karolinska Institutet, Stockholm, Sweden; 4 Department of Forensic Psychiatry, Niuvanniemi Hospital, University of Eastern Finland, Kuopio, Finland

**Keywords:** Parkinson’s disease, Antidepressant, Incidence, Epidemiology, Cohort studies

## Abstract

**Background:**

Antidepressant use is more common in people with Parkinson’s disease (PD), but it is unknown when this difference emerges.

**Methods:**

We studied the incidence of antidepressant use in six-month periods from 10 years before to 15 years after PD diagnosis in the nationwide register-based Finnish Study on Parkinson’s disease (FINPARK). This study included 20,456 community dwellers with clinically verified PD diagnosed during 1996–2015 and 140,291 matched comparison persons.

**Results:**

Altogether 44.3% of people with PD initiated antidepressants, compared to 25.0% of people without PD. The difference was largest 6 months before PD diagnosis (incidence rate ratio 5.28, 95% CI 4.80–5.80; 9.02 and 1.68 initiations/100 person-years in people with and without PD, respectively). The difference emerged already 7 years before the diagnosis and remained above the comparison group for most of the study period.

**Conclusions:**

Persons with PD may have symptoms that require antidepressant treatment years before and after diagnosis. The symptoms needing antidepressant treatment may be clinical signs of possible PD and they should be considered as a need to assess clinical status in person diagnosed with PD*.*

**Supplementary Information:**

The online version contains supplementary material available at 10.1186/s12877-021-02145-6.

## Background

The average age when Parkinson’s disease (PD) is diagnosed is approximately 65–70 years [1]. Therefore, due to population aging, the prevalence of age-related disease including Parkinson’s disease is expected to rise. Although PD is characterised by the motor symptoms, nonmotor symptoms are also common [2]. These symptoms range from functional somatic symptoms such as constipation, to sleep disorders and to psychological symptoms, anxiety and mood disorders, including major and minor depression as well as clinically relevant depressive symptoms and dysthymia [3–6]. They can occur years before PD diagnosis [3, 4] and precede the motor symptoms [6].

Studies on people diagnosed with PD have demonstrated higher prevalence of mood disorders in comparison to general population [2, 7–9]. Depression is more frequent in people with PD, although there is substantial variation in the estimated prevalence in people with PD (range 2.7–90%) [7, 10–12]. This variation likely stems from differences between study populations, diagnosis and definition of depression and statistical measures used in individual studies [7]. According to a systematic review [7], the prevalence was lower in population-based studies than studies conducted in outpatient, inpatient or nursing home settings. In addition for major depressive disorder according to DSM criteria, studies using structured or semistructured interview have reported higher prevalence than those without a structured interview [7]. Anxiety is also common among persons with PD, and it can occur independently or concomitantly with depression [8, 9].

Prevalence and incidence of antidepressants after initiation of dopaminergic antiparkinson drugs [13, 14] or inpatient or outpatient hospital visit with PD diagnosis [15] has been investigated. These studies demonstrated higher prevalence of antidepressant use among antiparkinson drug users than nonusers [13]. Antiparkinson drug users were also more likely to initiate antidepressants in comparison to nonusers of antiparkinson drugs [14]. One study compared the incidence of antidepressant use in cohort with hospitalisation with PD diagnosis to a cohort with hospitalisation with osteoarthritis diagnosis and showed that the incidence was higher in the PD cohort [15]. Due to the design of these previous studies (any dopaminergic antiparkinson drug use or admission to secondary healthcare with PD as the main diagnosis as proxy for PD, and the applied timeframe for assessing antidepressant incidence) it is unknown when the difference emerges in relation to PD diagnosis, and how long the difference remains.

To summarise, previous studies have shown higher prevalence of nonmotor symptoms, including possible reasons for antidepressant initiation, and higher incidence and prevalence of antidepressants in people with hospital admission for PD or PD drug users. However, it is unknown when the difference emerges in relation to PD diagnosis. To illustrate this, we investigated the incidence of antidepressant use in six-month time windows from 10 years before to 15 years after PD diagnosis in a nationwide cohort, and compared the incidence to a matched cohort without PD.

## Methods

The nationwide register-based Finnish Study on Parkinson’s disease register-based FINPARK study includes 22,189 people who received clinically confirmed PD diagnosis during the years 1996–2015 and were community-dwelling at the time of diagnosis.

People with PD diagnosis were identified from the Special Reimbursement Register maintained by the Social Insurance Institution of Finland (SII). Originally, 29,942 people eligible for reimbursement of anti-Parkinson drugs were identified, but as these drugs can also be used for other reasons, we excluded those who did not have ICD-10 code for PD (G20) recorded in the Special Reimbursement Register (*n* = 1244), those who were < 35 years old at the time of PD diagnosis (*N* = 53) and those who had diagnoses whose symptoms may be confused with PD (*n* = 6456) within 2 years of PD diagnosis, which lead to a cohort of 22,189 people. The exclusion diagnoses are listed in Supplementary Table 1. These people were excluded, as diagnosis of PD and its differential diagnostics is challenging, and false diagnoses are common in the early phase [16, 17]. The proportion of excluded people (25.9%) in our study is within the range of estimated proportion of false diagnoses [16, 17].

The application for special reimbursement includes anamnesis of the patient and description of the characteristic clinical features of PD including bradykinesia, rigidity and tremor. These applications are centrally reviewed in the SII. Special reimbursement for PD medications is granted if predefined criteria for PD diagnosis are fulfilled and diagnoses must be confirmed by a neurologist. Diagnosis of PD was based on United Kingdom Parkinson’s Disease Society Brain Bank’s criteria [18].

An age (+/− 1 year), sex and region-matched comparison cohort was identified from the SII database covering all residents. The index date was the date of PD diagnosis for the matched referent. The comparison people were not allowed to have purchases of PD medication (Anatomical Therapeutic Chemical classification ATC code N04) or the reimbursement code ever before the index date or 12 months after and during the diagnosis month of the referent people with PD. They also had to remain alive and community-dwelling during the month of index date. The diagnosis-based exclusion criteria of comparison people was otherwise similar to that of the PD cohort, but dementia due to PD (ICD-10 F02.3) was added to list of exclusion criteria, leaving altogether 148,009 comparison people. Dementia due to PD was added to ensure exclusion of people with PD from the comparison cohort.

Incidence of antidepressant use was investigated from 10 years before to 15 years after the PD diagnosis. Data on antidepressant purchases during 1995–2016 were gathered from the Prescription Register, which contains data on reimbursed medication purchases. Antidepressants were defined as ATC class N06A (Supplementary Table 2), and further categorized as selective serotonin reuptake inhibitors (SSRIs), tricyclic antidepressants (TCAs), serotonin–norepinephrine reuptake inhibitors (SNRIs), mirtazapine, and other antidepressants. Incident users were identified with a one-year washout-period, starting 11 years before PD diagnosis. For those diagnosed before or during the year 2005, year 1995 was used as a one-year washout-period. People who purchased antidepressants during the washout period, those who were hospitalised for > 50% of the washout or hospitalised for the last 90 days of washout were excluded (Supplementary Figure 1). Only the first initiation of antidepressant use after washout period was included in the analysis. Hospitalisation data were obtained from the Care Register for Health Care.

Data on comorbidities since 1972 until the index date were obtained from the Special Reimbursement register: asthma or chronic obstructive pulmonary disease (code 203), cardiovascular diseases including chronic heart failure (201) hypertension (205), coronary artery disease (206, 213, 280) and rheumatoid arthritis and connective tissue diseases (code 202). Diabetes was defined as special reimbursement code 103 or purchase of antidiabetics (ATC A10, excluding guar gum A10BX01). History of depression (discharge diagnosis or diagnosis in outpatient visit in specialized health care) was identified from the Care Register for Health Care using ICD-82960, 3004, 3011; ICD-9: 2961, 2968, 3011, 3004; ICD-10 F32-F34, F38-F39.

### Statistical analyses

The incidence rate (IR) of antidepressant initiations per 100 person-years was calculated for every six-month period starting from 10 years before and ending 15 years after the index date. The IRs were compared with Poisson regression between persons with PD and their comparison persons and reported as incidence rate ratios (IRR) with 95% confidence intervals (CI). As the Prescription register does not contain data on drugs used in hospitals or public nursing homes, people with 120 or more hospital days within a six-month period were excluded from that period.

The follow-up ended on initiation of antidepressant use, death, end of study follow-up (15 years after PD diagnosis) or end of data linkage (31.12.2016), whichever occurred first. In addition, comparison people who received PD diagnosis during the follow-up (*n* = 79) were censored on the date of diagnosis. Altogether 75 of these comparison people were diagnosed with PD during 1996–2015 so they were also included in the PD group. The results were similar after excluding these 75 people from the comparison group (data available from the corresponding author by request). The main analyses included all people, regardless of year of PD diagnoses but we performed sensitivity analysis on initiation of any antidepressant for those who had at least 10 years of purchase data prior to PD diagnosis (PD diagnosed in 2006–2015).

Characteristics of antidepressant initiators and non-initiators, and initiators with and without PD were compared with the chi-squared test for categorical variables and with *t*-test for the continuous variables. The analyses were performed using Stata MP 14.0.

## Results

The final study sample included 20,546 people with and 140,291 people without PD. During the study period, antidepressant initiation was more common among people with PD (*n* = 9091, 44.3%) compared to people without PD (*n* = 35,083, 25.0%). Women were more likely to initiate antidepressants in both groups. Association between baseline age and antidepressant initiation was not clinically significant, but people with PD were 1 year younger when they initiated antidepressants compared with persons without PD (Table 1). Depression was the only comorbidity that was more common among initiators with PD in comparison to initiators without PD (10.2% vs. 7.6%). In persons with and without PD, the most frequently initiated antidepressant group was SSRIs. SSRIs and mirtazapine together covered 81.0% of all initiations among users with PD and 78.7% among users without PD.

**Table 1:** Comparison of incident antidepressant initiators and noninitiators with and without PD. Comorbidities and university hospital district represent data on index date (date of Parkinson’s disease diagnosis)

	Parkinson’s disease *n* = 20,546			No Parkinson’s disease *n* = 140,291			
	Initiators (*n* = 9091)	Non-initiators (*n* = 11,455)	P*	Initiators (*n* = 35,083)	Non-initiators (*n* = 105,208)	P*	*P* **
Age at the beginning of follow-up mean (95% CI)	62.8 (62.5–63.0)	63.1 (63.0–63.3)	0.005	63.6 (63.5–63.7)	62.3 (62.3–62.4)	< 0.001	< 0.001
Sex (men) (n, %)	4332 (47.7)	7134 (62.3)	< 0.001	15,986 (45.6)	62,399 (59.3)	< 0.001	< 0.001
Comorbidities							
History of depression	924 (10.2)	152 (1.3)	< 0.001	2680 (7.6)	1163 (1.1)	< 0.001	< 0.001
Cardiovascular disease	3585 (39.4)	4414 (38.5)	0.19	14,937 (42.6)	38,377 (36.5)	< 0.001	< 0.001
Diabetes	1143 (12.6)	1490 (13.0)	0.36	4769 (13.6)	12,434 (11.8)	< 0.001	0.011
Asthma/COPD	713 (7.8)	768 (6.7)	0.002	3134 (8.9)	7166 (6.8)	< 0.001	0.001
Rheumatoid arthritis	309 (3.4)	375 (3.3)	0.62	1477 (4.2)	3662 (3.5)	< 0.001	< 0.001
University hospital district			< 0.001			< 0.001	0.25
Helsinki	2777 (30.6)	3169 (27.7)		10,458 (29.8)	30,480 (29.0)		
Kuopio	1698 (18.7)	2142 (18.7)		6593 (18.8)	19,245 (18.3)		
Oulu	1221 (13.4)	1675 (14.6)		4792 (13.7)	14,865 (14.1)		
Tampere	2164 (23.8)	2831 (24.7)		8561 (24.4)	25,753 (24.5)		
Turku	1193 (13.1)	1573 (13.7)		4578 (13.1)	14,231 (13.5)		
Other (Åland)	38 (0.4)	65 (0.6)		101 (0.3)	634 (0.6)		
Age at initiation (mean, 95% CI)	70.6 (70.4–70.9)			71.9 (71.8–72.0)			< 0.001
Number of purchased antidepressants							0.39
1	9028 (99.3)			34,868 (99.4)			
2	63 (0.7)			215 (0.6)			
Antidepressant group							
SSRIs	4899 (53.9)			16,973 (48.4)			< 0.001
Mirtazapine	2460 (27.1)			10,620 (30.3)			< 0.001
TCAs	958 (10.5)			4760 (13.6)			< 0.001
SNRIs	422 (4.6)			1711 (4.9)			0.35
Other	410 (4.5)			1208 (3.4)			< 0.001

*difference between initiators and noninitiators

**difference between initiators with and without PD

**Fig. 1:**
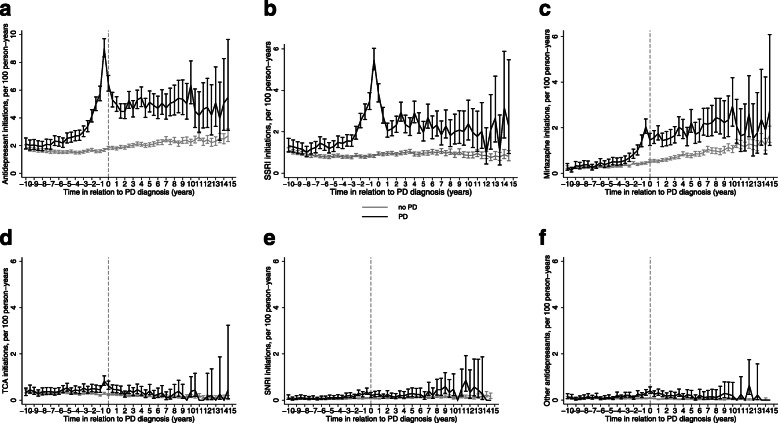
Incidence and 95% confidence intervals for **a** any antidepressant **b** selective serotonin release inhibitor (SSRI) **c** mirtazapine, **d** tricyclic antidepressant (TCA), **e** SNRIs and **f** other antidepressant use in relation to Parkinson’s disease (PD) diagnosis date. Black lines represent PD cohort and grey lines the comparison group

Among people with PD, the incidence was comparable to that of the comparison cohort until 7 years before PD diagnosis, when the difference began to emerge. The difference remained until the end of the follow-up, although the confidence intervals of the PD cohort widened, and overlapped with the comparison group at timepoint 13 years after PD diagnosis (Fig. 1a, Supplementary Table 3). Among people without PD the incidence of antidepressant initiation was approximately two per 100 person-years throughout the study period. The largest difference was observed 6 months before PD diagnosis (IRR 5.28, 95% confidence interval 4.80–5.55), with 9.02 initiations per 100 person-years among PD persons compared to 1.68 per 100 person-years among people without PD. Similar incidence peak and increase 7 years before PD diagnosis was observed in sensitivity analyses restricted to those with at least 10 year of purchase data prior to PD diagnosis (Supplementary Figure 2).

The incidence of SSRIs initiations followed similar trends with any antidepressant initiations, with the highest incidence observed 6 months before PD diagnosis (Fig. 1b). Similar incidence peaks, but at lower level were observed for mirtazapine and TCAs (Fig. 1c-d). There was a continuous upward trend in mirtazapine initiations in people with and without PD. The initiation of SNRIs and other antidepressants were less common, and the differences between people with and without PD were small (Fig. 1e-f).

## Discussion

Our study shows that the incidence of antidepressant use in people with PD began to increase already 7 years before the diagnosis, with peak occurring around 6 months before diagnosis. The incidence remained above the comparison group for over 10 years after PD diagnosis. These findings extend the previous evidence on higher prevalence of depression and depressive symptoms in people with PD [3–6], and studies showing higher prevalence and incidence of antidepressants in antiparkinson drug users [13, 14].

The added value of our study to previous studies on incidence and prevalence of antidepressant use in antiparkinson drug users [13, 14] or people with in- or outpatient hospital care with PD diagnosis [15] is providing more detailed information on temporal changes in antidepressant initiation in relation to PD diagnosis. A cross-sectional study compared the prevalence of antidepressant use between users and nonusers of dopaminergic antiparkinson drugs in one three-month time-window [13]. Thus, it did account for duration of antiparkinson drug use. The two studies which assessed incidence of antidepressant use after initiation of any dopaminergic antiparkinson drug [14] or hospital care (in- or outpatient) with PD as main diagnosis [15] reported the incidence for three different time windows (0-6 months, 6–12 months and over 1 year). Thus, it was not previously known when the difference emerges and how long it remains. In the two previous studies on the incidence of antidepressant use, the initiation rates peaked during the first 6 months after antiparkinson drug initiation or hospital admission with PD as main diagnosis [14, 15]. However, these studies began the follow-up from the initiation of antiparkinson drug or hospitalisation and due to these differences in study design they are not comparable with our study.

In addition, the use of dopaminergic antiparkinson drugs [13, 14] or secondary healthcare admission with PD as a main diagnosis [15] as a proxy for PD in these previous studies may complicate the comparison of our results to these previous studies. Diagnosis of PD is challenging, as indicated by the relatively large proportion of false diagnoses [16, 17]. Treatment trial on antiparkinson drugs is often a part of the diagnostic process, and these drugs can be used for other reasons, ranging from restless legs to rare cases of stopping milk production after labour. Therefore, previous studies excluded women aged 40 years or younger using bromocriptine and cabergoline [14] and restricted the studies to users of dopaminergic drugs [13, 14].

As our definition of PD was based on clinically confirmed diagnosis instead of antiparkinson drug use, it includes also those people who received the diagnosis regardless on whether or not they initiated the antiparkinson drugs on that specific date. People who likely initiated antiparkinson drugs for other reasons were not included. The proportion of people excluded due to possible misdiagnoses in our cohort is within the range of estimated proportion of false diagnoses [16, 17].

The increase in incidence rate already 7 years before PD diagnosis in our study is novel observation supported by the extending literature on non-motor symptoms including depression, anxiety and sleep disorders, which can occur years before PD diagnosis, and even before the motor symptoms [3–6]. According to a systematic review, history of depression is more common in people with PD [3], and a study based on the Health Improvement Network (THIN) database showed that depression, defined as Read code or antidepressant prescription was more common in people with PD already 5 years before diagnosis [4]. The higher incidence after PD diagnosis in our study may be explained by the high prevalence (40–50%) of depression in people with PD as depression is the most frequently reported neuropsychiatric disturbance in PD. [7] However, it should be noted that the CIs at the end of follow-up were wide because the number of eligible initiators had become small and in reality, the difference may remain longer.

SSRIs were the most frequently initiated antidepressants. This is expected, as according to a meta-analysis of different antidepressants groups, only SSRIs had significant effect on depression in PD [19]. In addition to mood disorders like depression [7], also anxiety and sleep disorders [3] as well peripheral neuropathic pain [20] are more common in people with PD, so these conditions may also explain the results. SNRIs and TCAs can be used to treat neuropathic pain, but their use was low in our study population. Sleep disorders may explain the increased initiation of mirtazapine during the follow-up.

We acknowledge that one limitation of our study is the lack of data on indication for antidepressant initiation. Thus, assessing the indications and acknowledging possibly changing indications during the disease course would be important in future studies. In our study, the prevalence of hospital-treated depression was 10.6% in antidepressant initiators with PD. Although this covers only the most severe cases of depression, it is unlikely that depression or depressive symptoms entirely explain the increase in incidence.

In conclusion, the initiation of antidepressants among people with PD became more common already 7 years before the PD diagnosis and continued to remain above comparison group more than 10 years after diagnosis. Persons with PD have neuropsychiatric symptoms such as depression that may require antidepressant treatment years before and after diagnosis. The symptoms needing antidepressant treatment may be clinical signs of possible PD and they should be considered as a need to assess clinical status in person diagnosed with PD*.*

## Supplementary Information

**Additional file 1: Supplementary Table 1.** Exclusion diagnoses for the PD cohort. **Supplementary Table 2.** Antidepressants and ATC-codes. **Supplementary Table 3.** Incidence rates of antidepressant initiation in people with and without Parkinson’s disease in different time periods. **Supplementary Figure 1.** Formation of study population. **Supplementary Figure 2.** Incidence of any antidepressant use in those with at least 10 years of purchase data before the index date (index date 1.1.2006–31.12.2015).

## Data Availability

The data that support the findings of this study are available from the corresponding author but restrictions apply to the availability of these data, and so they are not publicly available. Data are however available from the authors upon reasonable request and with permission of the register maintainers.

## Abbreviations

ATC: Anatomical therapeutic chemical classification
FINPARK: Finnish Study on Parkinson’s disease
IR: Incidence rate, IRR incidence rate ratio
PD: Parkinson’s disease
SII: Social Insurance Institution of Finland
SNRI: Serotonin–norepinephrine reuptake inhibitor
SSRI: Serotonin reuptake inhibitor
TCA: Tricyclic antidepressant
