# Supplementary material for: Incidence of antidepressant use among community dwellers with and without Parkinson’s disease – a nationwide cohort study
Source: BMC Geriatr. 2021 Mar 23;21:202. doi: 10.1186/s12877-021-02145-6 (PMC7986562; doi:10.1186/s12877-021-02145-6)

Supplementary material

**Incidence of antidepressant use among community dwellers with and without Parkinson`s disease – a nationwide cohort study**

Eerik Hentilä^1,2^; Miia Tiihonen^1,2^; Heidi Taipale^1,2,3,4^; Sirpa Hartikainen^1,2^; Anna-Maija Tolppanen^1,2^

^1^School of Pharmacy, Faculty of Health Sciences, University of Eastern Finland, P.O. Box 1627, 70211 Kuopio, Finland
²Kuopio Research Centre of Geriatric Care, University of Eastern Finland, P.O. Box 1627, 70211 Kuopio, Finland

^3^Department of Clinical Neuroscience, Karolinska Institutet, Stockholm, Sweden

^4^Department of Forensic Psychiatry, Niuvanniemi Hospital, University of Eastern Finland, Kuopio, Finland

Supplementary table 1. Exclusion diagnoses for the PD cohort.

| **Diagnosis** | **Data source and coding** |
| --- | --- |
| Secondary parkinsonism | Hospital discharge register ICD-9: 3321A; ICD-10: G21 |
| Other degenerative diseases of basal ganglia | Hospital discharge register ICD-9: 3330A; ICD-10: G23 |
| Dystonia | Hospital discharge register ICD-9: 3339X; ICD-10: G24 |
| Other extrapyramidal and movement disorders | Hospital discharge register ICD-9: 3331A, 3332A, 3333A, 3338X, 3339X; ICD-10: G25 |
| Huntington disease | Hospital discharge register ICD-9: 3334A; ICD-10: G10 |
| Hereditary ataxia | Hospital discharge register ICD-9: 3340A, 3341A, 3342A; ICD-10: G11 |
| Parkinsonism in diseases classified elsewhere | Hospital discharge register ICD-9: 0948X, 3321A; ICD-10: G22 |
| Systemic atrophies primarily affecting central nervous system in diseases classified elsewhere | Hospital discharge register ICD-9: 3318X, 3588X; ICD-10: G13 |
| Multiple sclerosis | Hospital discharge register ICD-9: 3400A; ICD-10: G35  Special reimbursement register codes 109,157,164,303,353 |
| Multi-system degeneration | Hospital discharge register ICD-9: 3378X; ICD-10: G90.3 |
| Other degenerative diseases of nervous system, not elsewhere classified | Hospital discharge register ICD-9: 3311A, 3312X, 3318X; ICD-10: G31 |
| Dementia | Hospital discharge register ICD-9: 3310A, 4378A, 3311A, 0461A, 3334A, 2941A, 2900A; ICD-10: F00-F03 excluding F02.3 |
| Alzheimer’s disease | Hospital discharge register ICD-9: 3310A; ICD-10: G30  Special reimbursement register code 307 together with ICD-10 code G30 |

Supplementary table 2. Antidepressants and ATC-codes

| Antidepressants | ATC-code (N06A) |
| --- | --- |
| Tricyclic antidepressants (TCAs) | N06AA |
| Selective serotonin reuptake inhibitors (SSRIs) | N06AB |
| Mirtazapine | N06AX11 |
| Serotonin–norepinephrine reuptake inhibitors (SNRIs): venlafaxine, milnacipran, duloxetine | N06AX16, N06AX17, N06AX21 |
| Other antidepressants: moclobemide, mianserin, trazodone, bupropion, reboxetine, agomelatine, vortioxetine | N06AG02, N06AX03, N06AX05, N06AX12, N06AX18, N06AX22, N06AX26 |

Supplementary table 3. Incidence rates of antidepressant initiation in people with and without Parkinson’s disease in different time periods.

|  | Incidence/100 person-years (95%CI) | | Incidence rate ratio (95%CI) |
| --- | --- | --- | --- |
| Time in relation to index date (years) | Parkinson’s disease | no Parkinson’s disease |  |
| -10 | 2.12 (1.76-2.56) | 1.91 (1.77-2.06) | 1.11 (0.91-1.36) |
| -9.5 | 2.02 (1.69-2.41) | 1.72 (1.6-1.86) | 1.17 (0.97-1.42) |
| -9 | 2.05 (1.72-2.44) | 1.7 (1.58-1.83) | 1.20 (1.00-1.45) |
| -8.5 | 1.97 (1.66-2.35) | 1.65 (1.54-1.78) | 1.19 (0.99-1.44) |
| -8 | 1.94 (1.63-2.31) | 1.65 (1.53-1.77) | 1.18 (0.98-1.42) |
| -7.5 | 2.1 (1.78-2.47) | 1.62 (1.5-1.74) | 1.30 (1.08-1.55) |
| -7 | 2.07 (1.75-2.43) | 1.55 (1.44-1.67) | 1.33 (1.11-1.59) |
| -6.5 | 2.33 (2-2.72) | 1.56 (1.45-1.68) | 1.49 (1.26-1.76) |
| -6 | 2.29 (1.96-2.66) | 1.54 (1.44-1.66) | 1.48 (1.25-1.75) |
| -5.5 | 2.18 (1.87-2.55) | 1.53 (1.42-1.64) | 1.43 (1.21-1.69) |
| -5 | 2.53 (2.2-2.91) | 1.54 (1.44-1.65) | 1.64 (1.40-1.92) |
| -4.5 | 2.59 (2.25-2.97) | 1.57 (1.47-1.68) | 1.64 (1.41-1.91) |
| -4 | 2.69 (2.35-3.08) | 1.5 (1.4-1.6) | 1.79 (1.54-2.08) |
| -3.5 | 2.72 (2.39-3.11) | 1.5 (1.4-1.6) | 1.81 (1.56-2.11) |
| -3 | 3.01 (2.65-3.42) | 1.56 (1.46-1.67) | 1.92 (1.67-2.22) |
| -2.5 | 3.38 (3.01-3.81) | 1.64 (1.54-1.75) | 2.05 (1.80-2.35) |
| -2 | 4.12 (3.71-4.59) | 1.7 (1.59-1.81) | 2.42 (2.14-2.74) |
| -1.5 | 4.99 (4.53-5.5) | 1.59 (1.49-1.7) | 3.11 (2.77-3.50) |
| -1 | 5.74 (5.24-6.28) | 1.6 (1.5-1.71) | 3.55 (3.17-3.96) |
| -0.5 | 9.02 (8.39-9.7) | 1.68 (1.58-1.79) | 5.28 (4.80-5.80) |
| 0 | 6.46 (5.92-7.05) | 1.84 (1.74-1.95) | 3.46 (3.12-3.84) |
| 0.5 | 5.29 (4.79-5.84) | 1.84 (1.74-1.95) | 2.84 (2.53-3.19) |
| 1 | 4.97 (4.47-5.52) | 1.8 (1.69-1.91) | 2.73 (2.42-3.08) |
| 1.5 | 4.47 (3.99-5.02) | 1.9 (1.79-2.02) | 2.33 (2.04-2.65) |
| 2 | 4.45 (3.95-5.02) | 1.86 (1.75-1.99) | 2.36 (2.06-2.70) |
| 2.5 | 5.26 (4.69-5.9) | 1.98 (1.86-2.11) | 2.62 (2.29-2.98) |
| 3 | 4.61 (4.06-5.25) | 2.02 (1.89-2.15) | 2.26 (1.96-2.61) |
| 3.5 | 5.29 (4.66-5.99) | 2.13 (1.99-2.27) | 2.44 (2.12-2.81) |
| 4 | 5.47 (4.81-6.23) | 2.09 (1.96-2.24) | 2.57 (2.22-2.97) |
| 4.5 | 4.67 (4.03-5.41) | 2.01 (1.87-2.16) | 2.29 (1.95-2.69) |
| 5 | 5.12 (4.42-5.94) | 2.06 (1.92-2.21) | 2.44 (2.08-2.88) |
| 5.5 | 4.9 (4.18-5.73) | 2.12 (1.97-2.28) | 2.27 (1.91-2.70) |
| 6 | 4.69 (3.95-5.56) | 2.23 (2.07-2.4) | 2.06 (1.71-2.48) |
| 6.5 | 5.06 (4.25-6.02) | 2.24 (2.08-2.42) | 2.21 (1.83-2.67) |
| 7 | 4.73 (3.9-5.72) | 2.47 (2.29-2.66) | 1.87 (1.52-2.30) |
| 7.5 | 5.04 (4.15-6.14) | 2.25 (2.08-2.45) | 2.18 (1.76-2.70) |
| 8 | 5.17 (4.21-6.35) | 2.4 (2.21-2.6) | 2.12 (1.70-2.64) |
| 8.5 | 5.42 (4.37-6.71) | 2.13 (1.94-2.33) | 2.48 (1.97-3.13) |
| 9 | 5.34 (4.24-6.73) | 2.36 (2.16-2.59) | 2.19 (1.71-2.81) |
| 9.5 | 5.03 (3.91-6.48) | 2.39 (2.17-2.62) | 2.07 (1.58-2.71) |
| 10 | 6.37 (5.02-8.1) | 2.24 (2.02-2.47) | 2.78 (2.14-3.61) |
| 10.5 | 4.48 (3.3-6.08) | 2.43 (2.2-2.69) | 1.79 (1.30-2.48) |
| 11 | 4.18 (2.97-5.88) | 2.61 (2.36-2.9) | 1.56 (1.09-2.23) |
| 11.5 | 4.62 (3.27-6.53) | 2.26 (2.02-2.54) | 2.01 (1.39-2.89) |
| 12 | 4.76 (3.31-6.85) | 2.43 (2.16-2.73) | 1.92 (1.31-2.82) |
| 12.5 | 4.2 (2.77-6.38) | 2.22 (1.95-2.53) | 1.85 (1.20-2.87) |
| 13 | 5.09 (3.39-7.67) | 2.31 (2.02-2.64) | 2.15 (1.40-3.31) |
| 13.5 | 3.96 (2.39-6.57) | 2.53 (2.21-2.89) | 1.53 (0.90-2.58) |
| 14 | 5.06 (3.1-8.27) | 2.47 (2.14-2.86) | 2.00 (1.20-3.34) |
| 14.5 | 5.47 (3.11-9.64) | 2.71 (2.3-3.2) | 1.99 (1.10-3.58) |

Supplementary Figure 1. Formation of study population

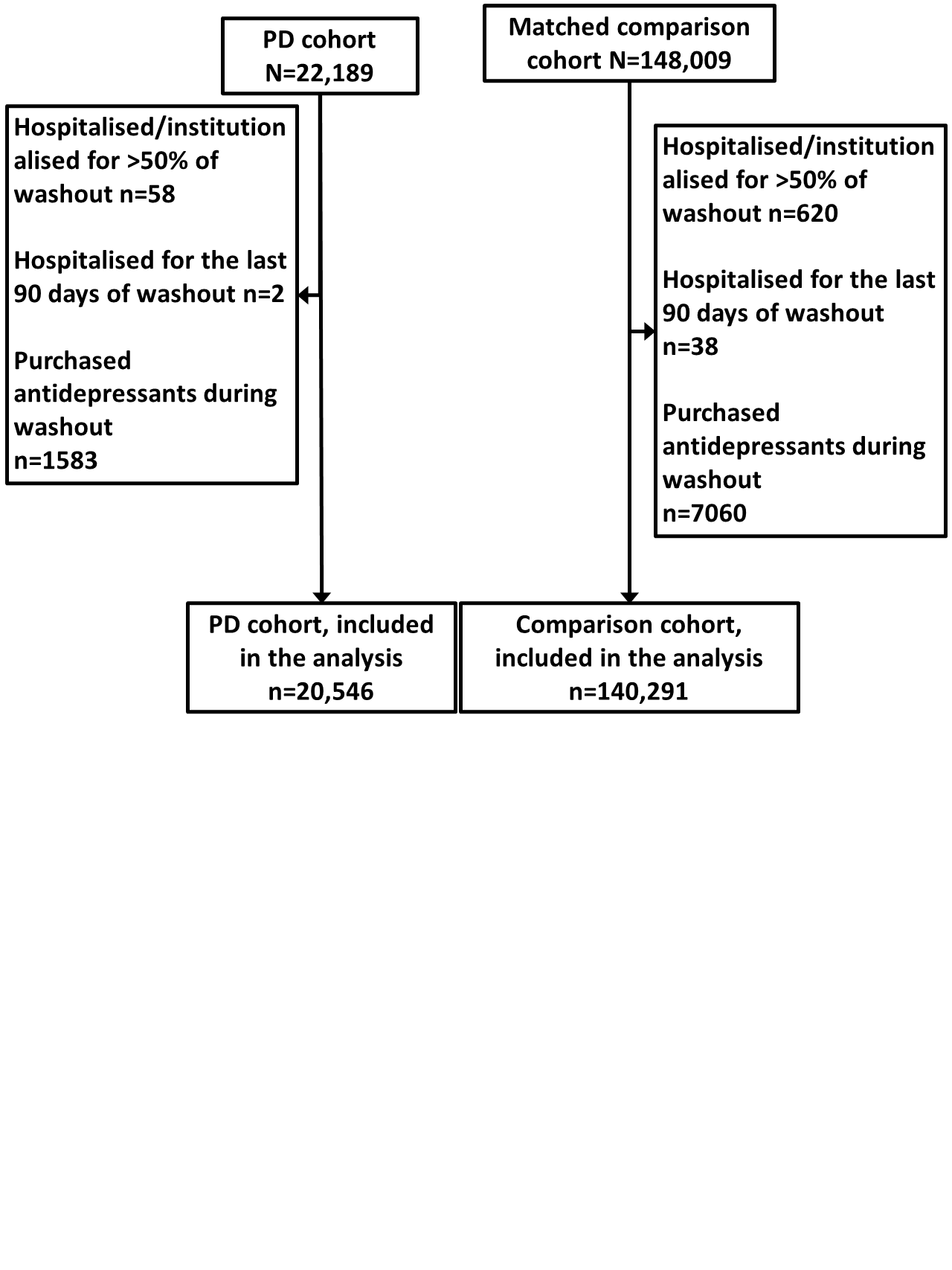


Supplementary Figure 2. Incidence of any antidepressant use in those with at least 10 years of purchase data before the index date (index date 1.1.2006-31.12.2015)
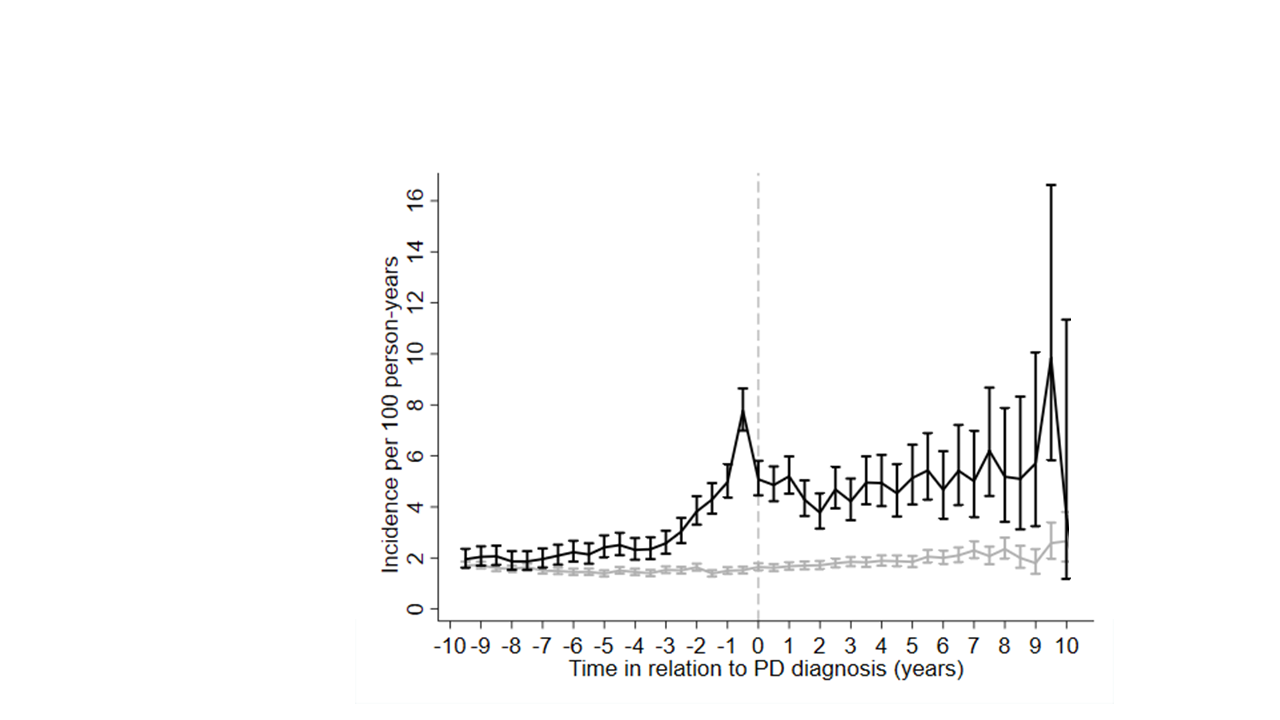

Supplement: Supplementary file 1 — Additional file 1: Supplementary Table 1. Exclusion diagnoses for the PD cohort. Supplementary Table 2. Antidepressants and ATC-codes. Supplementary Table 3. Incidence rates of antidepressant initiation in people with and without Parkinson’s disease in different time periods. Supplementary Figure 1. Formation of study population. Supplementary Figure 2. Incidence of any antidepressant use in those with at least 10 years of purchase data before the index date (index date 1.1.2006–31.12.2015). [file 12877_2021_2145_MOESM1_ESM.docx]
